# Genetic diversity and haplotype structure of the *CelTOS* gene in *Plasmodium falciparum* from Jazan Province, Saudi Arabia: implications for vaccine development and malaria elimination

**DOI:** 10.3389/fcimb.2025.1660506

**Published:** 2026-01-28

**Authors:** Hani Alothaid, Fatimah Alghnnam, Arwa A. Al-Qahtani, Abdullah Al Marzan, Mashael Abu Alola, Marie Fe F. Bohol, Fatimah Alhamlan, Mohammed I. Shafeai, Fuad H. Rudiny, Ali M. Motaen, Ahmed A. Al-Qahtani

**Affiliations:** 1 Department of Basic Medical Sciences, Faculty of Applied Medical Sciences, Al-Baha University, Al−Baha, Saudi Arabia; 2 Department of Infection and Immunity, Research Centre, King Faisal Specialist Hospital and Research Centre, Riyadh, Saudi Arabia; 3 Department of Family Medicine, College of Medicine, Imam Mohammad Ibn Saud Islamic University (IMSIU), Riyadh, Saudi Arabia; 4 Department of Data Science, Toxicology Society of Bangladesh, Dhaka, Bangladesh; 5 Department of Microbiology and Immunology, School of Medicine, Alfaisal University, Riyadh, Saudi Arabia; 6 Sabya General Hospital, Sabya, Saudi Arabia

**Keywords:** *CelTOS*, genetic diversity, malaria, molecular epidemiology, *Plasmodium falciparum*, seasonal variation, vaccine development

## Abstract

**Background:**

Malaria remains a persistent health challenge in southern Saudi Arabia, particularly along the border with Yemen. Understanding the genetic diversity and population structure of *Plasmodium falciparum* antigens is essential for tracking transmission dynamics and informing vaccine design. This study characterises population genetics of the Cell-Traversal Protein for Ookinetes and Sporozoites (*CelTOS*) gene in Jazan Province, a region of active transmission and frequent cross-border movement.

**Methods:**

We analysed 202 high-quality *P. falciparum CelTOS* coding sequences from 201 patients in Jazan. Diversity indices and neutrality tests were estimated in DnaSP, and demographic history was assessed using mismatch-distribution models (SSD, raggedness). Gene-wide dN/dS (ω) was derived from MEGA pairwise distances, and recombination was evaluated using PHI and GARD. Recombination-aware codon-based models (SLAC, FEL, MEME, and FUBAR) were implemented via Datamonkey. Population structure was examined using pairwise F_ST_, AMOVA, principal coordinate analysis (PCoA), and PERMANOVA. Haplotype networks were stratified by nationality, parasite density, region, and season, and parasitaemia correlates were explored using multivariable regression.

**Results:**

The 726-bp *CelTOS* alignment contained 18 segregating sites and 27 haplotypes (Hd = 0.921; π = 0.0059). Gene-wide ω was ≈0.36, consistent with predominant purifying selection, with only a few codons showing episodic diversifying selection. The mismatch distribution was unimodal (SSD = 0.029; raggedness = 0.0487), and recombination tests indicated low-level intragenic recombination (PHI p = 1.44 × 10^−5^; Rm = 4). Pairwise F_ST_ values were essentially zero, and PERMANOVA showed a weak, non-significant regional structure. Dominant *CelTOS* haplotypes were shared across nationality, density, and regional and seasonal strata. In the main logistic model (PD4 vs. PD1–PD3), age (OR 1.03; 95% CI 1.00–1.06) and non-Saudi nationality (OR 2.21; 95% CI 1.02–4.81) showed modest associations with very high parasitaemia.

**Conclusions:**

*CelTOS* diversity in Jazan is characterised by high haplotype but low nucleotide diversity within a largely panmictic, recombining parasite population. Extensive haplotype sharing across demographic and ecological gradients underscores strong gene flow, supporting cross-border, year-round control strategies and highlighting *CelTOS* as a relevant marker for vaccine development and molecular surveillance in this elimination setting.

## 1 Introduction

Malaria remains a significant public health challenge in the Kingdom of Saudi Arabia, particularly in the southwestern Jazan Province bordering Yemen. Despite considerable progress in malaria control efforts, with a 98% reduction in indigenous cases between 2000 and 2015 (Organization, 2023), the region continues to experience persistent transmission of *Plasmodium falciparum*, the most virulent malaria parasite species (Al-Mekhlafi et al., 2011). The ongoing cross-border movement between Saudi Arabia and Yemen, coupled with favourable climatic conditions for vector breeding, has contributed to the persistence of malaria in this region (Snow et al., 2013; Coleman et al., 2014).

The Jazan Province is considered the most malaria-endemic region in Saudi Arabia, accounting for more than 70% of the country’s malaria burden (Alzahrani et al., 2017). The area is characterised by diverse ecological settings, with varying altitudes, rainfall patterns, and human population demographics across its 10 administrative districts (Hawash et al., 2019). These factors create heterogeneous transmission dynamics that may influence parasite genetic diversity and population structure (Wesolowski et al., 2018). Previous studies have documented significant variations in malaria prevalence and incidence across different localities within Jazan, with higher rates typically observed in areas closer to the Yemeni border (Memish et al., 2014; Al-Mekhlafi et al., 2021).

The cell-traversal protein for ookinetes and sporozoites (*CelTOS*) is a micronemal protein expressed in motile stages of the malaria parasite and plays a crucial role in cell traversal of both mosquito midgut and vertebrate host cells (Kariu et al., 2006; Espinosa et al., 2017). This protein has emerged as a promising vaccine candidate due to its critical function in parasite infectivity and its relatively conserved nature across *Plasmodium* species (Bergmann-Leitner et al., 2010). Immunisation with recombinant *CelTOS* has demonstrated significant protection against sporozoite challenge in multiple animal models (Bergmann-Leitner et al., 2011), and anti-*CelTOS* antibodies from naturally exposed individuals have shown inhibitory activity against parasite invasion (Shekalaghe et al., 2014).

Genetic polymorphism in malaria antigens represents a major challenge for developing effective vaccines, as it enables parasites to evade host immune responses (Neafsey et al., 2015). Studies from various endemic regions have revealed different patterns of *CelTOS* genetic diversity, with some populations showing high conservation (Mehrizi et al., 2008) while others exhibit notable polymorphisms (Lumkul et al., 2018). The extent and distribution of these genetic variations can be influenced by multiple factors including transmission intensity, host immunity, and parasite population history (Barry and Arnott, 2014; Bei et al., 2015). Comprehensive characterisation of genetic diversity in candidate vaccine antigens such as *CelTOS* in endemic regions is therefore crucial for designing broadly protective vaccines (Draper et al., 2018).

Haplotype network analysis is a powerful tool for understanding parasite population genetics and molecular epidemiology (Rodrigues et al., 2018). By examining the relationships between genetic variants and their geographical distribution, haplotype networks can reveal patterns of parasite gene flow, population subdivision, and selective pressures (Branch et al., 2011). Furthermore, correlating haplotype distributions with host factors (such as age, sex, and nationality) and parasite characteristics (such as parasite density) can provide insights into the dynamics of host–parasite interactions and potential determinants of clinical outcomes (Auburn et al., 2012; Chenet et al., 2012).

In border regions like Jazan Province, where human migration significantly impacts malaria epidemiology, understanding the relationship between parasite genetic diversity and patient nationality becomes particularly important (Fola et al., 2018). Previous studies in other border regions have demonstrated distinct haplotype clustering associated with patient origin, reflecting separate parasite populations maintained through human movement (Lo et al., 2017). Similarly, parasite density has been linked to specific genetic variants in various Plasmodium antigens, potentially indicating differences in parasite fitness or virulence (Mharakurwa et al., 2013; Escalante et al., 2015).

This study aimed to investigate the genetic diversity of the *CelTOS* gene in *P. falciparum* isolates from the Jazan Province of Saudi Arabia, with a specific focus on haplotype distribution patterns across geographical locations, patient demographic characteristics, and clinical parameters. We collected samples from different localities across the province to provide a comprehensive representation of the parasite population structure in this endemic region. By characterising the patterns of nucleotide diversity and identifying potential selection pressures acting on this gene, our findings will contribute valuable information for malaria vaccine development strategies. Additionally, by analysing the relationship between *CelTOS* haplotypes and factors such as geographical location, patient nationality, parasite density, and seasonal variations, we aim to enhance our understanding of *P. falciparum* population dynamics and transmission patterns in the Saudi–Yemeni border region, which could inform targeted malaria control interventions.

## 2 Materials and methods

### 2.1 Ethical considerations

This study was reviewed and approved by the Ethics Review Committee of the King Fahad Central Hospital (KFCH), Jazan, Saudi Arabia (Approval No. 041). All procedures adhered to the ethical principles outlined in the Declaration of Helsinki (1975). Written informed consent was obtained from all participants; For minors, consent was provided by legal guardians. All personal identifiers were removed, and deidentified participant information was stored on a password-protected computer accessible only to authorised research personnel.

### 2.2 Study design, patients, and sample collection

A total of 201 patients with microscopically confirmed *P. falciparum* infection were enrolled from public health clinics across 10 regions of Jazan Province, including Al-Ardah, Bani-Malik, Abu-Arish, Baish, Sabya, Jazan, Al-Darb, Al-Ahad, Sametah, and Al-Twal. Finger-prick blood samples were collected on filter paper following a positive rapid diagnostic test (RDT). Thick and thin blood smears were prepared and examined by light microscopy after Giemsa staining to confirm parasitaemia and parasite morphology. Demographic and clinical metadata (age, sex, nationality, subregion, season of collection, and parasite density class) were recorded at enrolment using a standardised data sheet (**Supplementary File 1**).

Samples showing fungal contamination, inadequate DNA yield, or poor sequencing chromatogram quality were excluded. After quality control, 201 patient samples yielded 202 high-quality *CelTOS* sequences, which formed the input for all population-genetic and statistical analyses.

### 2.3 DNA extraction

Genomic DNA was extracted from dried blood spots using the DNeasy Blood and Tissue Kit (Qiagen GmbH, Hilden, Germany) according to the manufacturer’s instructions. Eluted DNA was quantified spectrophotometrically, stored at −20°C, and served as template for PCR amplification.

### 2.4 PCR amplification of the *CelTOS* gene

Amplification was performed using a nested PCR strategy to enhance sensitivity and specificity. Outer primers were used in the first amplification round, and inner primers targeted smaller fragments for the second round. All primers were designed in Primer3 (v4.1.0) with default settings; M13 tails were incorporated to facilitate downstream Sanger sequencing. Primer sequences are listed in **Table 1**.

**Table 1:** Primers used for amplification and sequencing of the *CelTOS* gene.

Primer name	Primer sequence
Round1_For-1	ATG TAC ATC TCC AAA CAA CAA A
Round1_Rev-1	AAC ACT CAG CCA ATC TTT TAT T
Round2_For-1.A	GTA AAA CGA CGG CCA GT GCC AAA TTA GCA CAC ACA TA
Round2_Rev-1.A	CAG GAA ACA GCT ATG ACC CAA CGG ACA CAA TTC TTC AT
Round2_For-2.B	GTA AAA CGA CGG CCA GT ATG GTC TCT GCT AAA TCA TCA
Round2_Rev-2.B	CAG GAA ACA GCT ATG ACC AAA ACA GTG AAC TCA TGC ATA T

### 2.5 PCR reaction mix and thermal cycling conditions

First-round PCR was conducted in 30-µL reactions containing 5 µL of DNA template, 1× GoTaq Green Master Mix (Promega, Madison, WI, USA), and 0.5 µM of each outer primer (Round1For-1/Round1Rev-1). Cycling consisted of 95°C for 3 min; 45 cycles of 95°C for 30 s, 56°C for 45 s, and 72°C for 90 s, followed by 72°C for 5 min.

Nested PCR reactions (segments A and B) were performed in 30-µL reactions using 5 µL of first-round product, 1× GoTaq Green Master Mix, and 0.5 µM of each inner primer. Cycling consisted of 95°C for 3 min; 45 cycles of 95°C for 30 s, 52°C for 45 s, and 72°C for 90 s, followed by 72°C for 5 min. Amplicons were verified on 2% agarose gels and purified prior to sequencing.

### 2.6 Sanger sequencing and chromatogram quality filtering

Purified PCR products were bidirectionally sequenced using M13 forward and reverse primers. To minimise PCR-induced artefacts and sequencing errors, all samples were independently amplified and Sanger-sequenced in duplicate, and 20% of the cohort was randomly selected for a third round of amplification using freshly prepared reaction mixes to assess reproducibility. Chromatograms were examined manually in Chromas (v2.6.6) and BioEdit, and aligned traces were evaluated for peak quality, baseline noise, and mixed peaks. Sequence variants were accepted only when consistently observed in both forward and reverse directions and reproducible across replicate reactions. Secondary peaks ≥25% of the primary peak height were assigned IUPAC ambiguity codes. Samples with low signal intensity or persistent multilocus double peaks suggestive of polyclonal infections, frame shifts, or premature stop codons were excluded from downstream analyses.

### 2.7 Sequence assembly and alignment parameters

Chromatograms were assembled into consensus sequences using SeqMan Pro (v15.3.0, DNASTAR Lasergene), and FASTA files were exported using EditSeq (v15.3.0). Multiple-sequence alignments were performed using ClustalW implemented in MegAlign Pro (v15.3.0) with the following parameters; gap opening penalty: 15 (nucleotide), gap extension penalty: 6.66, transitions/transversions ratio: default, and delay divergent sequences: 30%.

Stop codons and frameshifts were inspected to detect artefactual insertions or sequencing errors. Sequences containing ambiguous regions longer than five nucleotides were excluded.

### 2.8 Statistical and population genetic analyses

A suite of statistical and population genetic analyses was applied to assess genetic diversity, evolutionary pressures, demographic patterns, and associations with clinical and demographic variables. The analyses included mismatch distribution, selection tests, recombination detection, haplotype network construction, population-structure estimation (F_ST_, AMOVA), and regression modelling to evaluate the influence of demographic and geographic factors.

### 2.9 Haplotype and diversity analyses

We generated a codon-aware alignment of the P. falciparum CelTOS open reading frame from the curated consensus sequences using MACSE v2.07 (Multiple Alignment of Coding SEquences) and then imported the resulting nucleotide alignment into MEGA v12 for manual inspection and trimming. The final codon-based alignment (202 sequences; 726 ungapped sites) used for all diversity and neutrality analyses is provided as **Supplementary File 2** (CelTOS_Codon_Alignment.fasta).

Global and group-specific diversity indices were estimated in DnaSP v6.12.03 (DNA Polymorphism module, “analysis at individual sites”, column-by-column mode). For the overall dataset and for predefined sequence sets (by region, season, and patient nationality), we calculated the number of segregating sites (S), the total number of mutations (Eta), the number of haplotypes (H), haplotype diversity (Hd) with its variance, nucleotide diversity (π) per site, and Watterson’s θ_w per site.

Sequence sets for the three Jazan subregions, two transmission seasons, and nationality strata were defined using the “Define Sequence Sets” option in DnaSP. The full numerical outputs for all groups are provided in **Supplementary File 3a** (DnaSP_Diversity_Neutrality_by_Group.xlsx), and the global summary of diversity and neutrality indices (S, H, Hd, π, θ_w, Tajima’s D, Fu’s Fs, Fu & Li’s D* and F*) is given in **Supplementary File 4a** (Summary of Genetic Diversity and Neutrality Parameters.xlsx).

### 2.10 Mismatch distribution analyses

To investigate demographic history, we analysed the distribution of pairwise nucleotide differences among *CelTOS* sequences. Site-wise diversity outputs from DnaSP v6.12.03 were exported and used to compute the mismatch distribution, the sum of squared deviations (SSD) between the observed and expected distribution under a sudden-expansion model, and Harpending’s raggedness index (r). All calculations followed the standard “sudden expansion” framework and assumed a constant mutation rate across sites.

**Figure 1:**
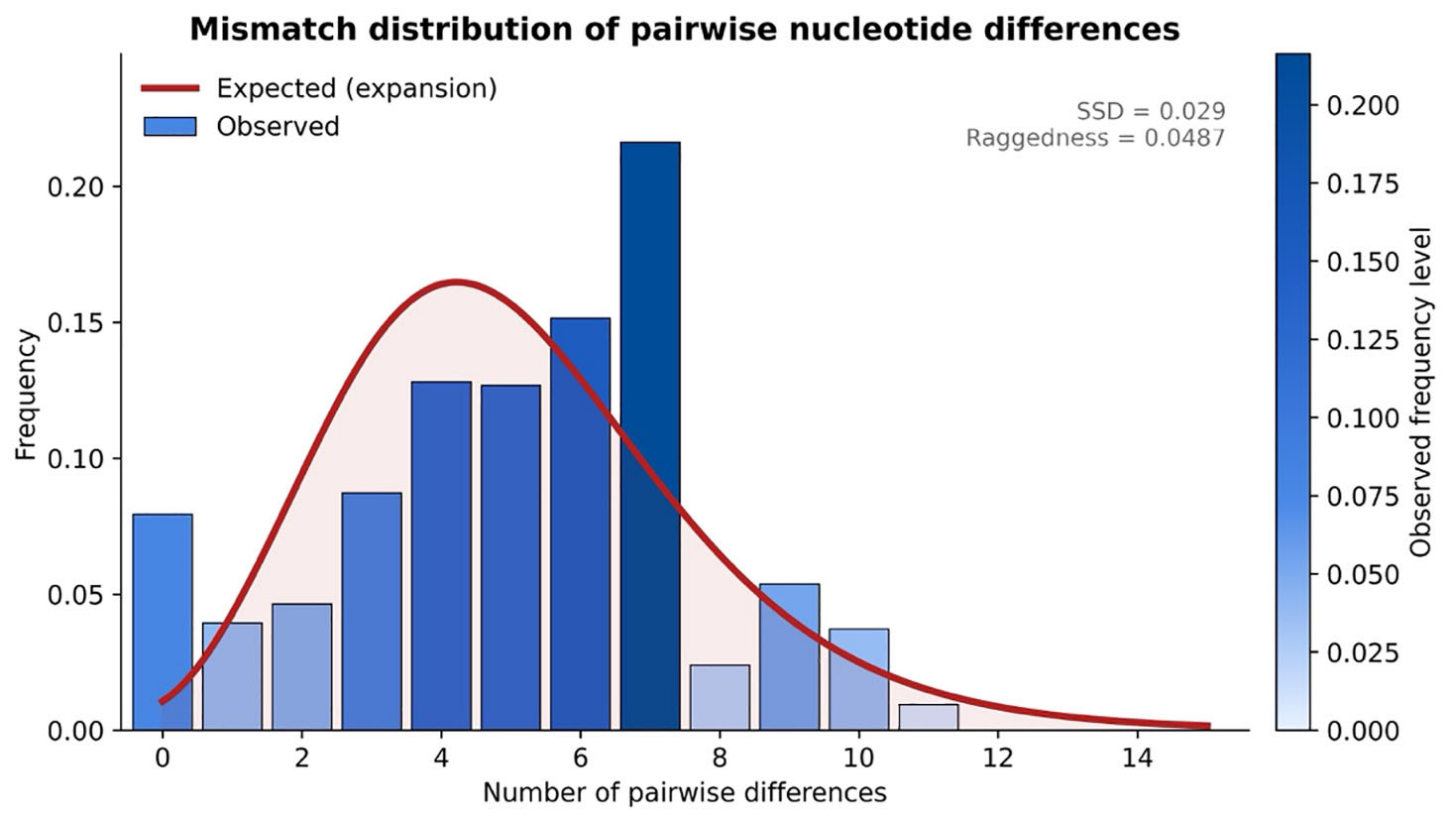
Mismatch distribution of pairwise nucleotide differences among *P. falciparum CelTOS* sequences. The observed distribution (blue) is broadly unimodal and lies close to the expected curve (red) under a sudden population-expansion model. Goodness-of-fit statistics: SSD = 0.029; raggedness index r = 0.0487.

The observed mismatch distribution and the smooth expected curve under demographic expansion were visualised using a custom Python script (**Figure 1**; mismatch.txt + figure1_mismatch.py; included in the Supplementary Python Scripts archive), which reads the DnaSP-formatted mismatch output (**Supplementary File 3b**; DnaSP_Mismatch_Data.xlsx) and generates a high-resolution figure and the corresponding SSD and raggedness values. This analysis is now described once in the Methods to avoid redundancy.

### 2.11 Synonymous and non-synonymous substitution rates

Gene-wide synonymous (dS) and non-synonymous (dN) substitution rates for *CelTOS* were estimated in MEGA v12 using the modified Nei–Gojobori method with Jukes–Cantor correction, the standard genetic code, uniform rates among sites, homogeneous patterns among lineages, and pairwise deletion of gaps and missing data. MEGA was used to export the full lower-triangular pairwise distance matrices for dN and dS.

To obtain a robust gene-wide estimate of ω (dN/dS), we did not average raw pairwise dN/dS ratios. Instead, we computed the mean dN and mean dS across all pairwise comparisons with dS ≥ 0.001 and then took their ratio, ω = mean(dN)/mean(dS). This filtering avoids extreme inflation of ω when dS is close to zero. The calculation was performed with a reproducible Python script (global_dnds.py; provided in the Supplementary Python Scripts archive), which parses the MEGA Excel exports (*CelTOS*_pairwise_dN.xlsx and *CelTOS*_pairwise_dS.xlsx) and reports mean dN, mean dS, and the resulting ω.

### 2.12 Geographic sub-analyses

To assess spatial variation, sequences were stratified by region. Regional diversity metrics were calculated when the post-QC sample size per region was ≥8. Regions with fewer than eight high-quality sequences were combined with geographically adjacent districts to preserve statistical power.

Subregional datasets were generated by stratifying samples according to collection locality. Diversity and neutrality indices (Hd, π, Tajima’s D, Fu & Li’s D*, and Fu & Li’s F*) were computed in DnaSP v6.12.03 under default parameters, and values were compared descriptively across geographic partitions.

### 2.13 Recombination analysis

Recombination was assessed using multiple approaches. The Phi test for recombination was conducted in SplitsTree v4.18.3. Minimum recombination events (Rm) were estimated using the Hudson method in DnaSP v6.12.03. Breakpoint analysis was performed using GARD implemented in HyPhy on the Datamonkey server. Tests were evaluated at α = 0.05. Evidence from these complementary methods was used to judge the extent of intragenic recombination and to interpret demographic and codon-based selection analyses; low-level recombination did not preclude use of the full alignment but was considered when interpreting haplotype structure and neutrality statistics.

### 2.14 Haplotype network construction

Haplotype networks were constructed using PopART v1.7 (Population Analysis with Reticulate Trees) employing the median-joining (MJ) algorithm with ϵ = 0. Gaps were treated as missing data, and IUPAC ambiguity codes were preserved. Node sizes were scaled proportionally to the number of identical sequences comprising each haplotype. Mutation steps separating haplotypes were inferred under parsimony and displayed along connecting edges. Degree centrality (number of direct node connections) was calculated to assess relative network connectivity.

### 2.15 Hierarchical AMOVA and pairwise F_ST_ estimates

Hierarchical AMOVA was performed in Arlequin v3.5 using 10,000 permutations to assess variance components among and within predefined groupings (region, season, and nationality). Significance of variance partitions was evaluated at α = 0.05. Pairwise F_ST_ values were calculated under the Tamura–Nei model, and negative values were interpreted as zero, reflecting sampling variance rather than biological similarity.

### 2.16 Association between clinical variables and parasitaemia

To explore whether basic host characteristics were associated with parasite density, we linked the *CelTOS* alignment to the clinical metadata compiled in **Supplementary File 1** (Traits.xlsx). For each patient, we considered: subregion (regions 1–3), age (years), sex (binary), and nationality (Saudi vs. non-Saudi), together with categorical parasite density. Parasitaemia at enrolment was recorded in four ordered classes (PD1–PD4), which we defined *a priori* as an ordinal severity scale (PD1 = lowest, PD4 = highest).

We first fitted an ordinary least-squares (OLS) linear model and several exploratory models (univariable OLS, ordinal logistic regression for PD1–PD4, and alternative binary cut-points) using Python 3.12 and the statsmodels package. These exploratory analyses are summarised in **Supplementary File 4b** (Regression_All_Models.xlsx) and were used to check robustness and avoid overinterpreting weak trends in a modest sample size.

For the main text, we prespecified a clinically interpretable binary outcome that distinguishes very high parasitaemia (PD4) from all lower categories (PD1–PD3). We then fitted a multivariable logistic regression model with high parasitaemia as the dependent variable and region (Region_num), age (years), sex (Sex_num), and nationality (Nat_num) as predictors:


logit{Pr(PD=4)}=β0+β1Region_num+β2Age+β3Sex_num+β4Nat_num.

Observations with missing or “Unknown” values in any predictor were excluded list-wise. Regression coefficients, standard errors, Wald z statistics, p-values, 95% confidence intervals, and odds ratios (OR) with 95% CI are reported in **Table 2**. This model corresponds to the sheet “Logit_PD4_vs_1to3_params” in Regression_All_Models.xlsx (**Supplementary File 4b**) and was selected because (i) it focuses on the most extreme density category (PD4), corresponding to the upper end of the “high parasitemia” spectrum used in descriptive analyses, and (ii) it showed the most consistent signals across related model specifications while avoiding overfitting.

**Table 2:** Multivariable logistic regression of very high parasitaemia (PD4) versus lower categories (PD1–PD3).

Variable	Coefficient	Std. error	z value	p-value	95% CI (lower)	95% CI (upper)	OR	OR 95% CI (lower)	OR 95% CI (upper)
Region	0.037	0.260	0.144	0.885	-0.471	0.546	1.038	0.625	1.725
Age	0.031	0.015	2.077	0.0378	0.002	0.059	1.031	1.002	1.061
Sex	0.244	0.499	0.489	0.625	−0.734	1.222	1.276	0.480	3.392
Nationality	0.795	0.396	2.007	0.045	0.019	1.572	2.214	1.019	4.814

The table shows regression coefficients (β), standard errors (SE), Wald z-statistics, p-values, 95% confidence intervals (CI) for β, and corresponding odds ratios (OR) with 95% CI. Age and nationality were independently associated with very high parasitaemia, whereas region and sex were not significant predictors.

### 2.17 Exclusion criteria summary

Samples were excluded if they met any of the following: insufficient DNA yield, poor bidirectional chromatogram quality, ambiguous peaks at >3 loci, failed replicate amplification, evidence of mixed infection (persistent multilocus heterozygosity).

### 2.18 Supplementary datasets and analysis files

All datasets and analysis outputs referenced in this article are provided as **Supplementary Files 1–7**, together with an accompanying archive of Python scripts. **Supplementary File 2** the final codon-aware CelTOS coding-sequence alignment used for all diversity, neutrality, mismatch, and recombination analyses. **Supplementary File 1** holds the individual-level clinical and demographic metadata linked to each sequence (age, sex, nationality, subregion, season, and parasite-density class) and underlies all regression models and stratified genetic analyses. **Supplementary File 3a** provides group-stratified DnaSP outputs for diversity and neutrality indices (by region, season, and nationality), and **Supplementary File 3b** contains the DnaSP mismatch-distribution export used to estimate the sum of squared deviations and Harpending’s raggedness index.

**Supplementary File 4a** collates the global diversity and neutrality summary statistics reported in the main text, whereas **Supplementary File 4b** documents the full set of exploratory and final regression models linking parasite density to demographic and geographic covariates. **Supplementary File 5a** (AMOVA_Summary.xlsx) presents summary variance components and Φ-statistics from the AMOVA, and **Supplementary File 5b** (AMOVA_Full_Results.xml) contains the complete Arlequin project file with all underlying AMOVA and pairwise FST outputs. **Supplementary File 6a** (Haplotype_Statistics.xlsx) tabulates CelTOS haplotype frequencies by region, season, nationality, and parasite-density class, and **Supplementary File 6b** (Haplotype_Definitions.txt) lists the nucleotide definitions of all 27 haplotypes. **Supplementary File 7** (Site_Level_Selection.xlsx) provides codon-level selection metrics from SLAC, FEL, MEME, and FUBAR analyses for each site across the CelTOS coding sequence.

### 2.19 Data availability and reproducibility

**Figure 2:**
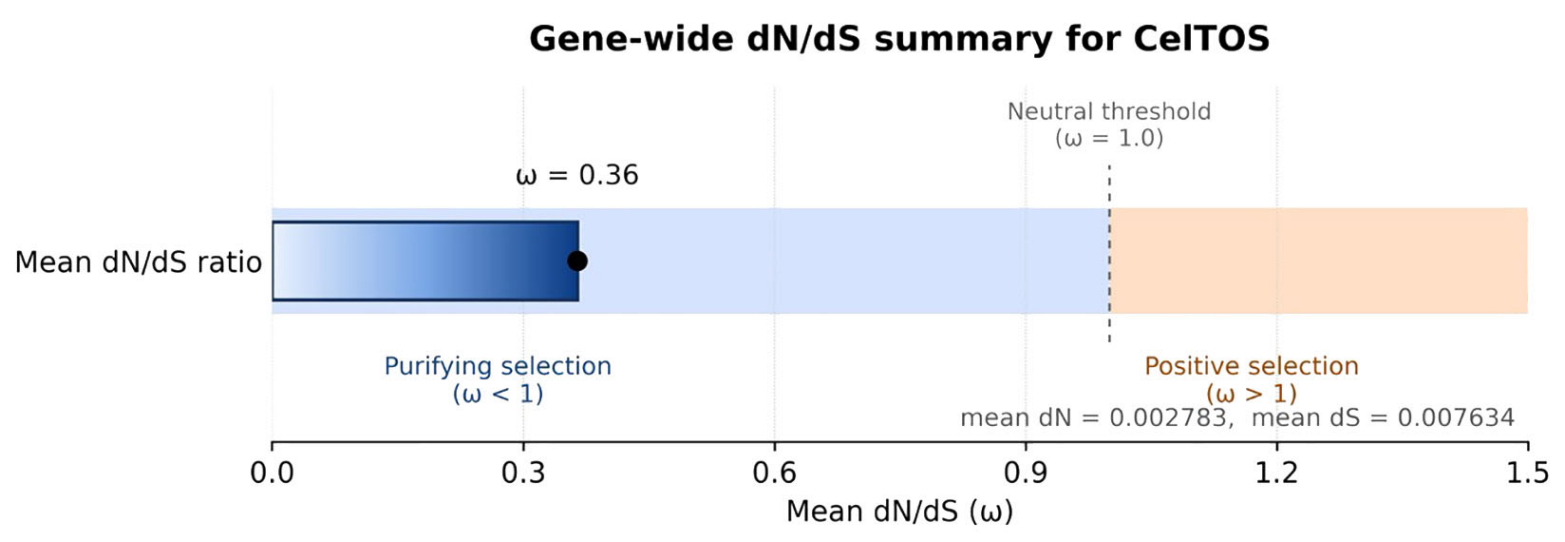
Gene-wide dN/dS ratio (ω) estimated from *CelTOS* coding sequences using the modified Nei–Gojobori method (Jukes–Cantor) in MEGA v12. The global ω of 0.36 is below the neutrality expectation of 1.0, indicating predominant purifying selection on the *CelTOS* protein, although codon-based models still identify a limited number of exposed sites under episodic diversifying selection (global_dnds.py).

**Figure 3:**
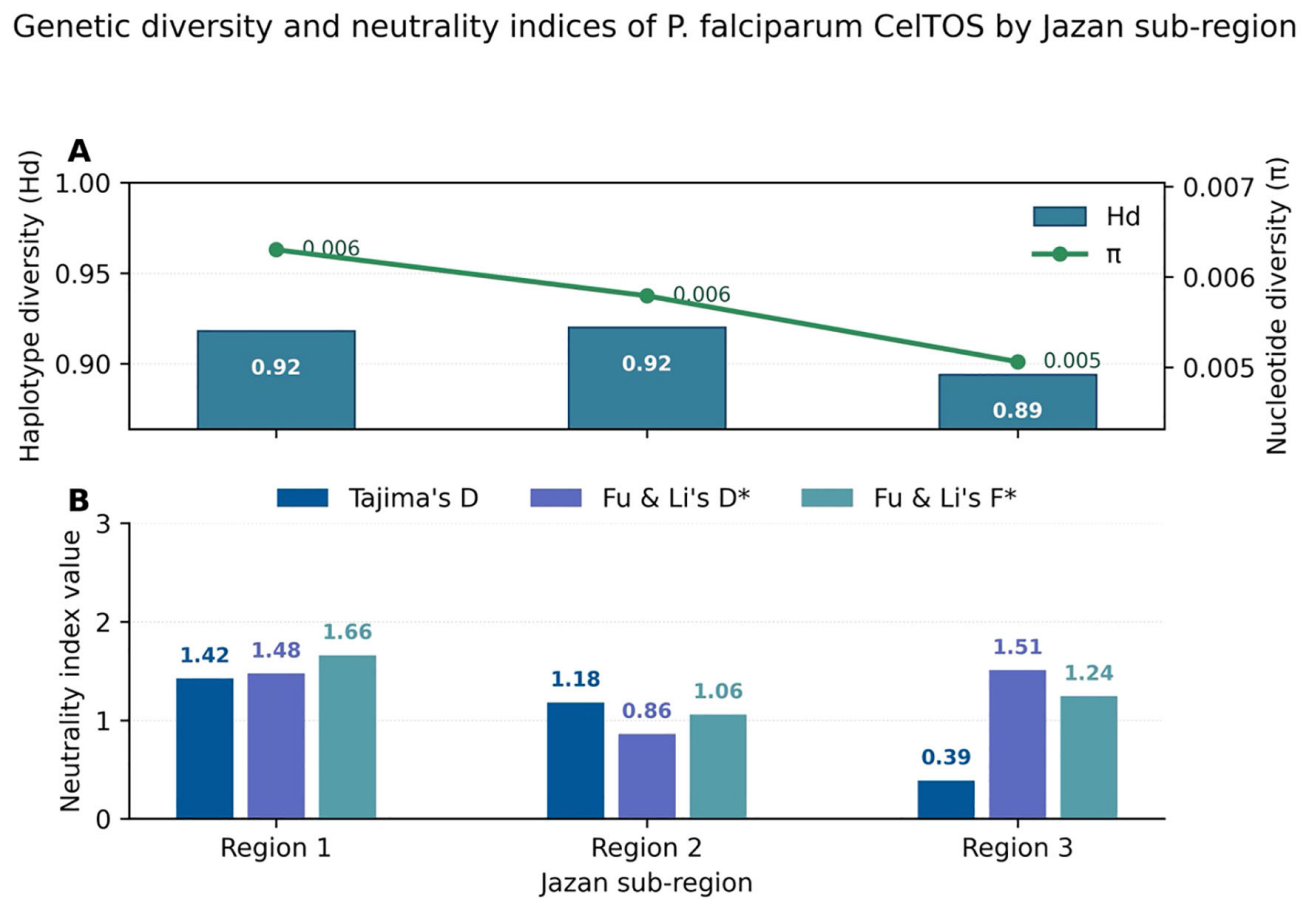
Genetic diversity and neutrality indices of *P. falciparum CelTOS* across three Jazan subregions. **(A)** Haplotype diversity (Hd, blue bars) and nucleotide diversity (π, green points with numeric labels) for each subregion. All three areas show high haplotype diversity (Hd ≈ 0.89–0.92) but low nucleotide diversity (π ≈ 0.005–0.006), indicating many closely related haplotypes within each locality. **(B)** Neutrality statistics (Tajima’s D, Fu & Li’s D*, Fu & Li’s F*) plotted on a common scale; the horizontal line at zero marks the expectation under mutation–drift equilibrium. All indices are positive and of similar magnitude across regions, suggesting mildly non-neutral but broadly comparable demographic histories.

**Figure 4:**
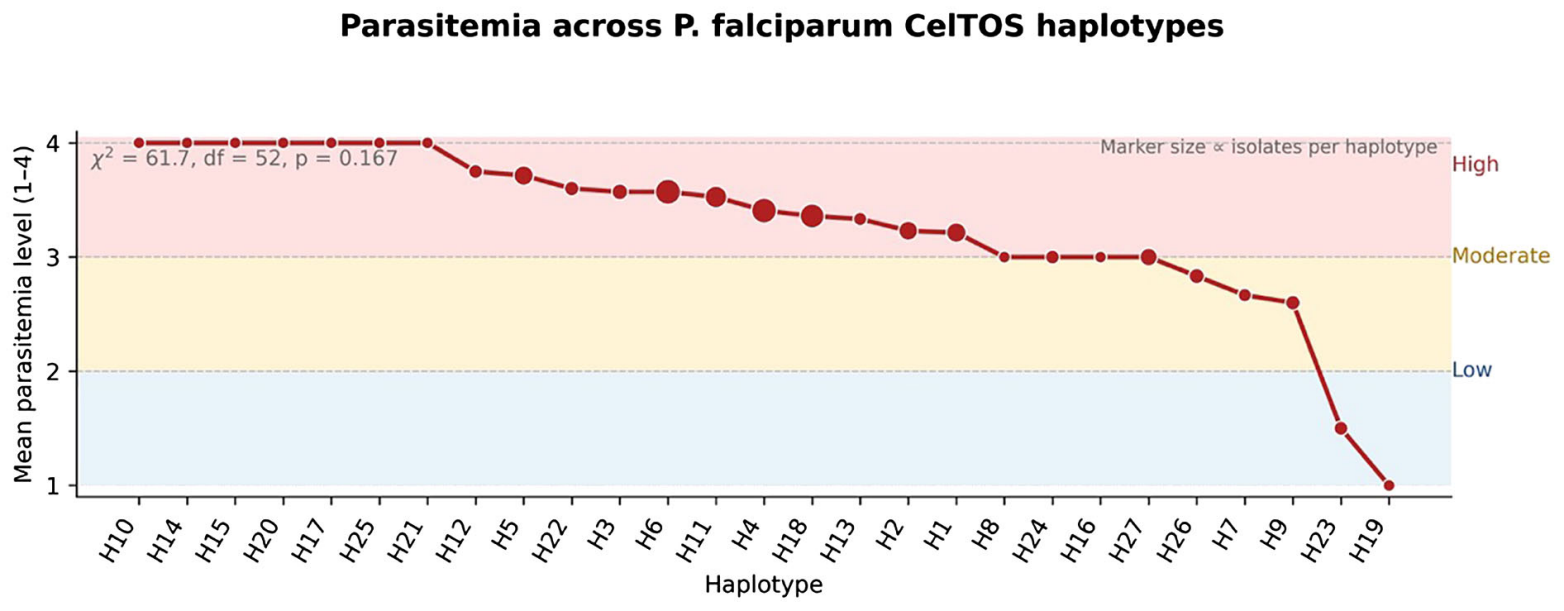
Parasitaemia across *P. falciparum CelTOS* haplotypes. Points show the mean parasitaemia level for each haplotype on an ordinal 1–4 scale corresponding to PD1–PD4, with marker size proportional to the number of isolates contributing to each mean. Background shading highlights approximate “low” (levels 1–2), “moderate” (around 3), and “high” (around 4) parasitaemia ranges. A global chi-square test for independence between haplotype and parasitaemia category found no strong evidence of heterogeneity (χ² = 61.7, df = 52, p = 0.167).

**Figure 5:**
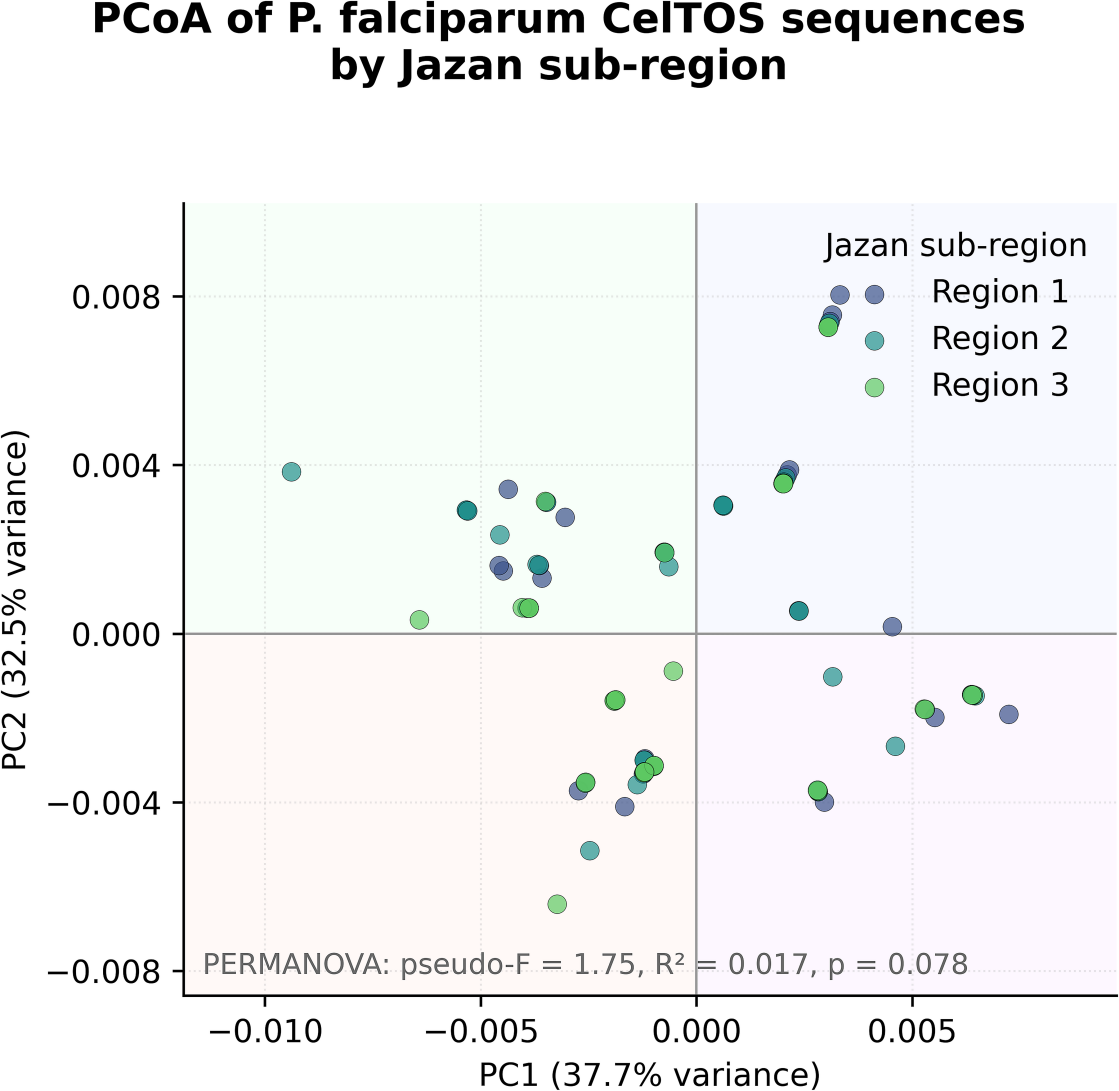
Principal coordinate analysis (PCoA) of *P. falciparum CelTOS* sequences by the Jazan subregion. The first two principal coordinates (PC1 and PC2) explain 37.7% and 32.5% of the genetic variance, respectively. Points are coloured by subregion (regions 1–3). Clusters show substantial overlap, and a PERMANOVA test for regional differences gives pseudo-F = 1.75, R² = 0.017, p = 0.078, consistent with weak and statistically non-significant geographic structure.

**Figure 6:**
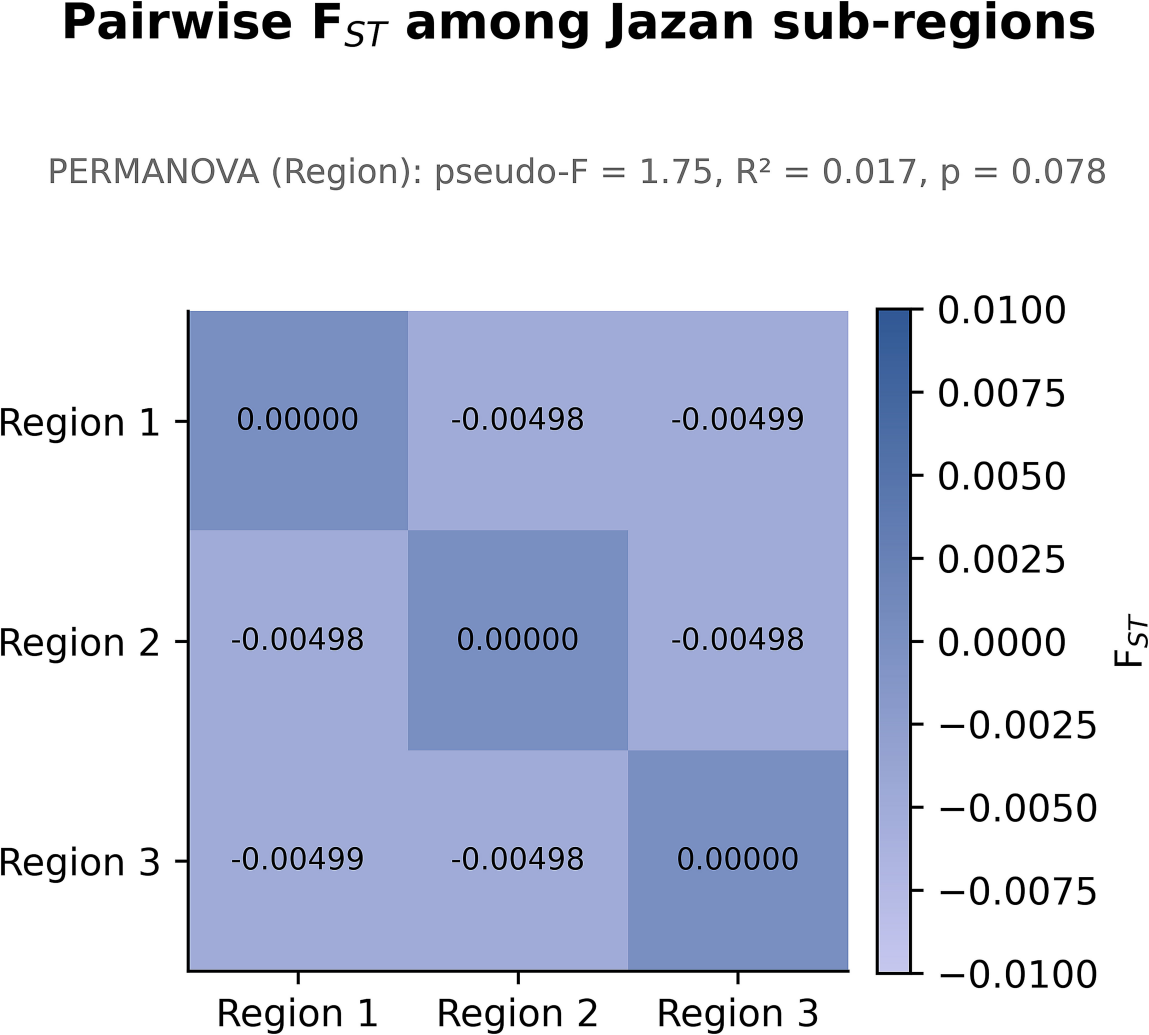
Pairwise F_ST_ among Jazan subregions. Heat map of pairwise F_ST_ estimates comparing *P. falciparum CelTOS* genetic differentiation among regions 1–3. The matrix is symmetrical with zeros on the diagonal (self-comparisons). All off-diagonal values are very close to zero (F_ST_ ≈ −0.005), and the colour scale is truncated to −0.01 to 0.01 to emphasise this near-absence of divergence. The PERMANOVA summary for region (pseudo-F = 1.75, R² = 0.017, p = 0.078) is shown above the plot and is concordant with the weak structure seen in the PCoA (**Figure 5**).

All core input data required to reproduce the analyses comprise the CelTOS codon alignment, the linked clinical and demographic traits file, and the accompanying Python scripts. The alignment and traits file are provided as **Supplementary Files 1, 2**, respectively, and the downstream numeric outputs are collated in **Supplementary Files 3a, b, 4a, b, 5a, b, 6a, b, 7**. All custom Python scripts used to generate **Figures 1**–**6**, to compute the gene-wide dN/dS ratio, to prepare AMOVA summaries, and to fit the regression models (including figure1_mismatch.py, figure2_dnds.py, figure3_diversity_neutrality.py, figure4_parasitemia_by_haplotype.py, figure5_pcoa.py, figure6_pairwise_Fst_regions.py, global_dnds.py and table3_regression.py) are bundled in the “Python Scripts” archive in the Supplementary Materials, together with the corresponding input files. These **Supplementary Files 1–7** and the Python Scripts archive are submitted with this manuscript and will accompany the published article.

In addition, A.A. Marzan maintains a version-controlled GitHub repository for the analysis scripts and supporting files used in this study to support reproducibility. The full set of materials is provided as Supplementary Material with the published article and is archived on Figshare with an assigned DOI.

## 3 Results

### 3.1 Patient demographics

A total of 201 patients infected with *P. falciparum* were enrolled in this study. The mean age of participants was 28.0 ± 13.9 years, with a median of 25.5 years (interquartile range [IQR]: 19.0–36.8 years). The cohort was predominantly male (72.1%, n = 145), whereas female participants accounted for 27.9% (n = 56).

Parasite density at enrolment was categorised into four World Health Organization (WHO)–aligned classes: PD1 (<1,000 parasites/µL), PD2 (1,000–9,999 parasites/µL), PD3 (10,000–99,999 parasites/µL), and PD4 (≥100,000 parasites/µL). Overall, 18.9% (n = 38) of patients were classified as PD1, 21.4% (n = 43) as PD2, 17.9% (n = 36) as PD3, and 41.8% (n = 84) as PD4 (**Table 3**). The spatial distribution of enrolled cases, and the full individual-level metadata used in downstream analyses are listed in **Supplementary File 1**.

**Table 3:** Patient characteristics at baseline parasitaemia.

Variables	Value
Age	
Mean ± SD	28.01 ± 13.95
Median (IQR)	25.50 (19.0–36.75)
Sex	
Male, n (%)	145 (72.14%)
Female, n (%)	56 (27.86%)
Parasite density	
Low (PD1) (<1,000 parasites/µL), n (%)	38 (18.90%)
Moderate (PD2) (1,000–9,999 parasites/µL), n (%)	43 (21.40%)
Moderate/high (PD3) (10,000–99,999 parasites/µL) n (%)	36 (17.91%)
High (PD4) (≥100,000 parasites/µL), n (%)	84 (41.79%)

### 3.2 Genetic diversity and neutrality analyses

The final molecular dataset used for population-genetic analyses comprised 202 P*. falciparum CelTOS* sequences from Jazan Province. After assembly quality control and codon-aware alignment, the final coding alignment comprised 242 codons (726 ungapped nucleotide sites) in an intact open reading frame. Analysis of this alignment in DnaSP v6.12.03 (“DNA Polymorphism” module, analysis at individual sites) identified 18 segregating sites (S), all of which were parsimony-informative, yielding 27 haplotypes in the full dataset. The overall haplotype diversity was Hd = 0.921 (variance ≈ 0.00005), indicating that two randomly drawn isolates are almost always different at *CelTOS*. By contrast, nucleotide diversity was low (π = 0.0059 per site), and Watterson’s θw was similarly modest (θw = 0.0035 per site), consistent with many distinct haplotypes separated by a small number of mutations.

Neutrality statistics for the global alignment showed a positive Tajima’s D (D = 1.77; 0.10 > p > 0.05), a modestly negative Fu’s Fs (Fs = –3.36), and positive Fu & Li’s D* and F* (D* = 1.43, F* = 1.76). These values do not provide strong evidence for a departure from mutation–drift equilibrium but are compatible with a slight excess of intermediate-frequency variants and a somewhat elevated haplotype count. The full set of diversity and neutrality indices by Jazan subregion, season, and nationality strata is provided in **Supplementary File 3a**, and the global summary is collated in **Supplementary File 4a**.

### 3.3 Mismatch distribution and demographic inference

The observed mismatch distribution of pairwise nucleotide differences across *CelTOS* sequences was unimodal and closely followed the distribution expected under a model of sudden demographic expansion (**Figure 1**). The sum of squared deviations (SSD) between the observed and modelled distributions was low (SSD = 0.029), and the raggedness index was similarly modest (r = 0.0487), indicating a smooth distribution with limited multimodality. These patterns are consistent with a parasite population that has experienced either a past expansion or sustained high gene flow, rather than long-term strong subdivision into isolated lineages.

The underlying calculations (mismatch classes, observed vs. expected frequencies, SSD, and raggedness) are provided in **Supplementary File 3b** and can be regenerated using the accompanying Python script (figure1_mismatch.py) supplied in the Supplementary Python Scripts archive.

### 3.4 Synonymous and non-synonymous substitution rates

Pairwise non-synonymous (dN) and synonymous (dS) distances for *CelTOS* were estimated in MEGA v12 using the modified Nei–Gojobori method with Jukes–Cantor correction. After excluding comparisons with negligible synonymous divergence (dS< 0.001), we obtained a mean dN of 0.0028 and a mean dS of 0.0076, corresponding to a global dN/dS ratio ω ≈ 0.36. This value is well below 1, indicating that *CelTOS* is subject to overall purifying (negative) selection at the gene level, rather than pervasive diversifying selection.

The distribution of pairwise dN and dS values, together with the gene-wide ω and its interpretation, is summarised in **Figure 2**. The full pairwise distance matrices and the Python script (global_dnds.py) used to derive the mean dN, mean dS, and ω are provided as part of the Supplementary Material, allowing the calculation to be reproduced exactly from the MEGA outputs.

Codon-based analyses performed with HyPhy nonetheless detected episodic diversifying selection at several codons using the MEME model (p< 0.1). Additional FEL and SLAC models identified a smaller subset of sites under positive selection. These selected residues mapped primarily to solvent-exposed or predicted immunogenic domains of *CelTOS*, suggesting localised immune-mediated amino-acid variation on a largely conserved functional backbone.

### 3.5 Genetic diversity and neutrality by the Jazan subregion

We next examined whether *CelTOS* diversity and neutrality statistics differed across the three Jazan subregions (Region 1–3; **Supplementary File 1**). As shown in **Figure 3**, haplotype diversity (Hd) was uniformly high across regions 1–3 (Hd ≈ 0.89–0.92), whereas nucleotide diversity (π) remained low in all subregions. The dual-axis display (bars for Hd, points/line for π) emphasises that each subregion harbours many distinct haplotypes separated by few nucleotide differences.

Neutrality indices for each region (Tajima’s D, Fu & Li’s D*, Fu & Li’s F*) are plotted on a common y-axis in **Figure 3** to avoid mixing statistics with different scales. All three subregions showed similarly positive values (Tajima’s D ≈ 0.4–1.5; Fu & Li’s D and F ≈ 0.8–1.7), indicating a mild excess of intermediate-frequency variants that is directionally consistent with the global analysis. Importantly, the magnitudes of these indices are comparable across regions and do not single out any subregion as having a uniquely extreme demographic signature. These results reinforce the view that the *CelTOS* locus is highly diverse but only weakly structured within Jazan, with most variation shared across regions. Detailed numerical values for each subregion are provided in **Supplementary File 3a**.

### 3.6 Parasitaemia distribution and clinical associations

To investigate potential associations between genetic variation and clinical presentation, we coded each parasitaemia class (PD1–PD4) as an ordinal score (1–4) and plotted the mean parasitaemia level per *CelTOS* haplotype (**Figure 4**). Most haplotypes had mean values within the upper (“high”) range, indicating that heavy infections were common across lineages, while a small subset (e.g., H23, H19) showed lower mean levels. However, a chi-square test of independence between haplotype and parasitaemia category did not reach conventional statistical significance (χ² = 61.7, df = 52, p = 0.167), suggesting that we could not detect a strong haplotype-specific effect on peripheral parasite density with the current sample size.

### 3.7 Population structure and differentiation analyses

A principal coordinate analysis (PCoA) was performed to visualise the spatial distribution of *CelTOS* genetic variation across the three Jazan subregions (**Figure 5**). The first two principal coordinates explained 37.7% and 32.5% of the total variance, respectively, capturing most of the observed genetic structure. Although some visual clustering by region is apparent, the subregional point clouds overlap extensively. In keeping with this pattern, PERMANOVA indicated only weak, statistically non-significant differences among regions (pseudo-F = 1.75, R² = 0.017, p = 0.078).

Overall, the PCoA therefore suggests at most very modest geographic differentiation in the *CelTOS* dataset. These results align with the modest clustering seen in the PCoA and near-zero F_ST_ values, and the full AMOVA outputs are provided in **Supplementary File 5b**. Summary AMOVA variance components and Φ-statistics are collated in **Supplementary File 5a**, and the full AMOVA outputs, including all pairwise F_ST_ matrices, are provided in **Supplementary File 5b**.

Genetic differentiation among the three subregions was assessed using pairwise F_ST_ estimates and hierarchical analysis of molecular variance (AMOVA) (**Figure 6**). The updated pairwise F_ST_ values were essentially zero for all regional comparisons (F_ST_ ≈ −0.005), indicating no measurable genetic divergence among regional parasite populations. Slightly negative F_ST_ estimates are interpreted as artefacts of sampling variance rather than evidence of “extra” similarity. Consistent with these findings, AMOVA showed that the vast majority of genetic variation (>95%) resides within regions, with only a very small and non-significant among-region component (10,000 permutations). Together with the PCoA and PERMANOVA results (pseudo-F = 1.75, R² = 0.017, p = 0.078), these patterns support a largely panmictic *P. falciparum* population within Jazan, with at most very weak geographic structuring at the *CelTOS* locus.

### 3.8 Multivariable logistic regression model for high parasitaemia

To assess whether basic demographic characteristics predicted very high parasite densities, we modelled the odds of PD4 vs. PD1–3 using multivariable logistic regression (Methods; **Table 2**). In the final model (n = 142 complete cases), age and nationality showed statistically significant associations with high parasitaemia, whereas region of residence and sex did not.

Each additional year of age was associated with a modest increase in the odds of very high parasitaemia (OR = 1.03; 95% CI 1.00–1.06; p = 0.038). Non-Saudi nationality was associated with approximately a two-fold higher odds of PD4 compared with Saudi patients (OR = 2.21; 95% CI 1.02–4.81; p = 0.045). Subregion (Region_num) and sex (Sex_num) had odds ratios close to unity with wide confidence intervals crossing 1 and non-significant p-values.

Overall, the regression analysis suggests that older age and non-Saudi nationality may modestly increase the likelihood of very high parasitaemia at presentation, but the effect sizes are small and should be interpreted cautiously given the limited sample size. Exploratory models using alternative parasitaemia cut-points (e.g., PD3–4 vs. PD1–2) and ordinal logistic regression yielded qualitatively similar conclusions and are summarised in **Supplementary File 4b**.

### 3.9 Recombination screening

To evaluate whether intragenic recombination might bias population-genetic inferences, the *CelTOS* alignment was analysed using multiple complementary approaches. The pairwise homoplasy index (Phi) test implemented in SplitsTree provided statistically significant evidence for recombination (p = 1.438 × 10^−5^), rejecting the null hypothesis of strictly clonal evolution. The Hudson recombination parameter estimated in DnaSP was R = 34.9 per gene (0.0399 between adjacent sites), and the minimum number of recombination events was R_m_ = 4, based on the four-gamete test. These results indicate that at least several historical recombination events have occurred within the ~726-site *CelTOS* fragment.

In contrast, the GARD analysis in HyPhy did not identify any well-supported recombination breakpoints, which may reflect limited power given the short locus length and modest number of polymorphic sites. Taken together, we interpret these findings as evidence for low-level recombination superimposed on an otherwise largely clonal background. This level of recombination is unlikely to invalidate our diversity and neutrality estimates but should be borne in mind when interpreting haplotype structure and demographic signals.

### 3.10 Haplotype network analysis of the *CelTOS* gene by patient nationality

A total of 27 distinct *CelTOS* haplotypes were identified from *P. falciparum* isolates collected across Jazan Province. Sample sizes per haplotype ranged from singletons (n = 1; e.g., Hap_5, Hap_15, Hap_17, Hap_21, Hap_22, Hap_23, Hap_24–27) up to n = 28 isolates for Hap_2 and Hap_6, with Hap_4 (n = 25), Hap_7 (n = 19), Hap_1 and Hap_3 (each n = 14), and Hap_10 (n = 13) forming an intermediate high-frequency group. This long-tailed distribution—with a few very common types and many rare ones—indicates high haplotype richness with a pronounced core–periphery structure.

**Figure 7:**
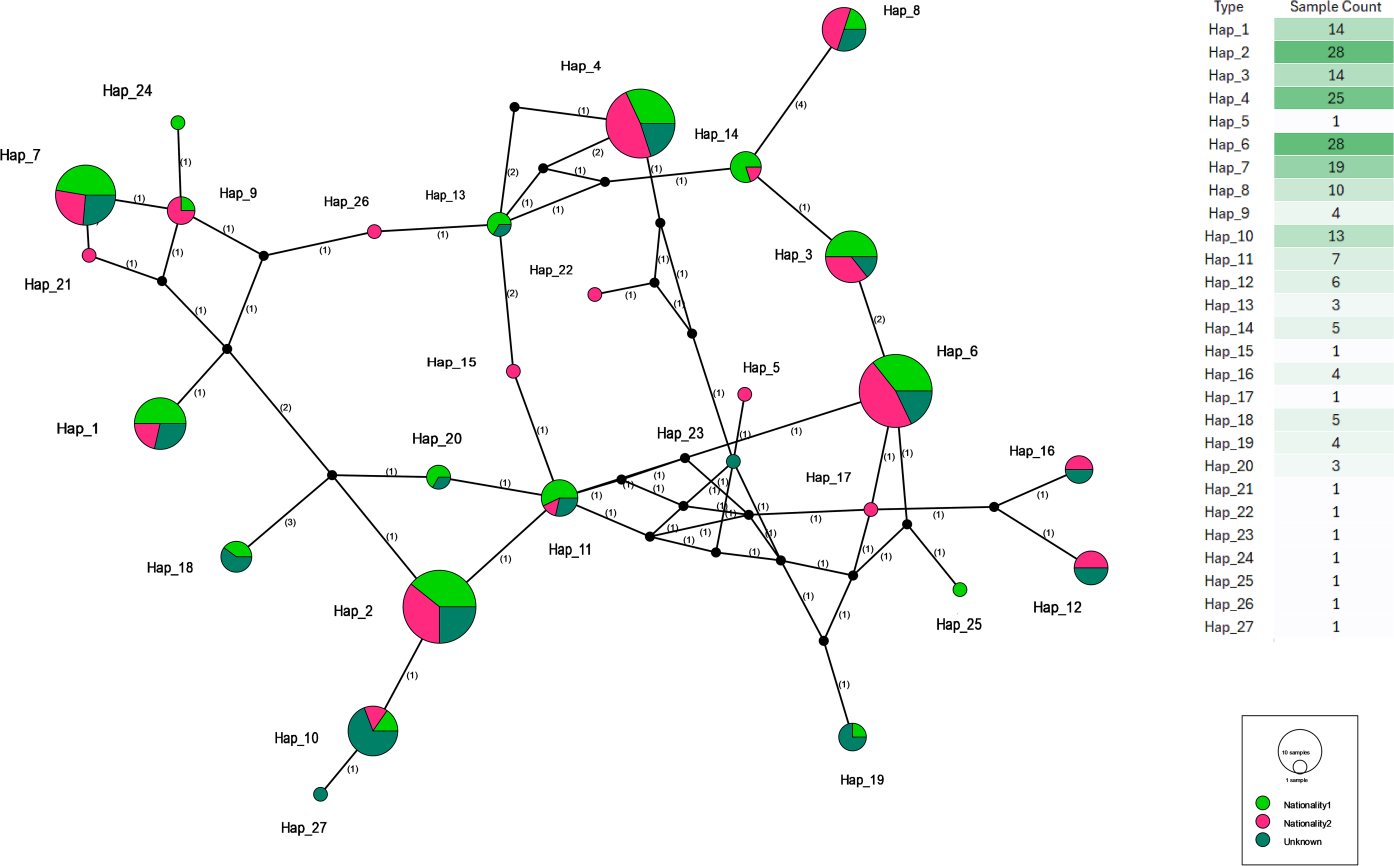
Haplotype network of *P. falciparum CelTOS* gene sequences categorised by patient nationality. Each node represents a unique haplotype (n = 27), with node size proportional to haplotype frequency. Pie-chart colours indicate patient nationality: pink = Saudi nationals, green = non-Saudi nationals, dark green = unknown or missing nationality data. Nucleotide differences are indicated along the edges. Shared haplotypes between nationality groups suggest widespread circulation and gene flow across populations in Jazan Province. Counts underlying the pies are in **Supplementary File 6a** (Nationality); haplotype sequences are in **Supplementary File 6b**.

Isolates were stratified by patient nationality into Nationality1, Nationality2, and Unknown. In the median-joining network (**Figure 7**), the most frequent, centrally located haplotypes are also the most nationality-diverse. For example, Hap_2, Hap_4, Hap_6, Hap_7, Hap_1, Hap_3, Hap_10, and Hap_11 all contain a mixture of Nationality1 and Nationality2, with several of them also including isolates of unknown nationality. Hap_6 and Hap_2, in particular, show substantial wedges from all three nationality categories, indicating extensive mixing of host backgrounds within these lineages.

By contrast, many of the peripheral, low-frequency haplotypes are “narrow” and dominated by a single nationality. Several singletons (e.g., Hap_5, Hap_15, Hap_17, Hap_21, Hap_22, and Hap_24–27) are represented exclusively by one nationality category, and a few slightly larger nodes (e.g., Hap_16 and Hap_19) also show strong nationality skew. These restricted haplotypes likely reflect recent or localised transmission events that have not yet spread widely across host populations.

Taken together, the strong nationality mixing within Hap_2, Hap_4, Hap_6, Hap_7, and other core haplotypes, combined with the short mutational distances linking rarer types back to these cores, supports a scenario of substantial gene flow between nationality groups. Nationality-biased haplotypes exist but are numerically rare and peripheral, suggesting limited parasite compartmentalisation by patient origin.

### 3.11 Haplotype network analysis of the *CelTOS* gene by parasite density

**Figure 8:**
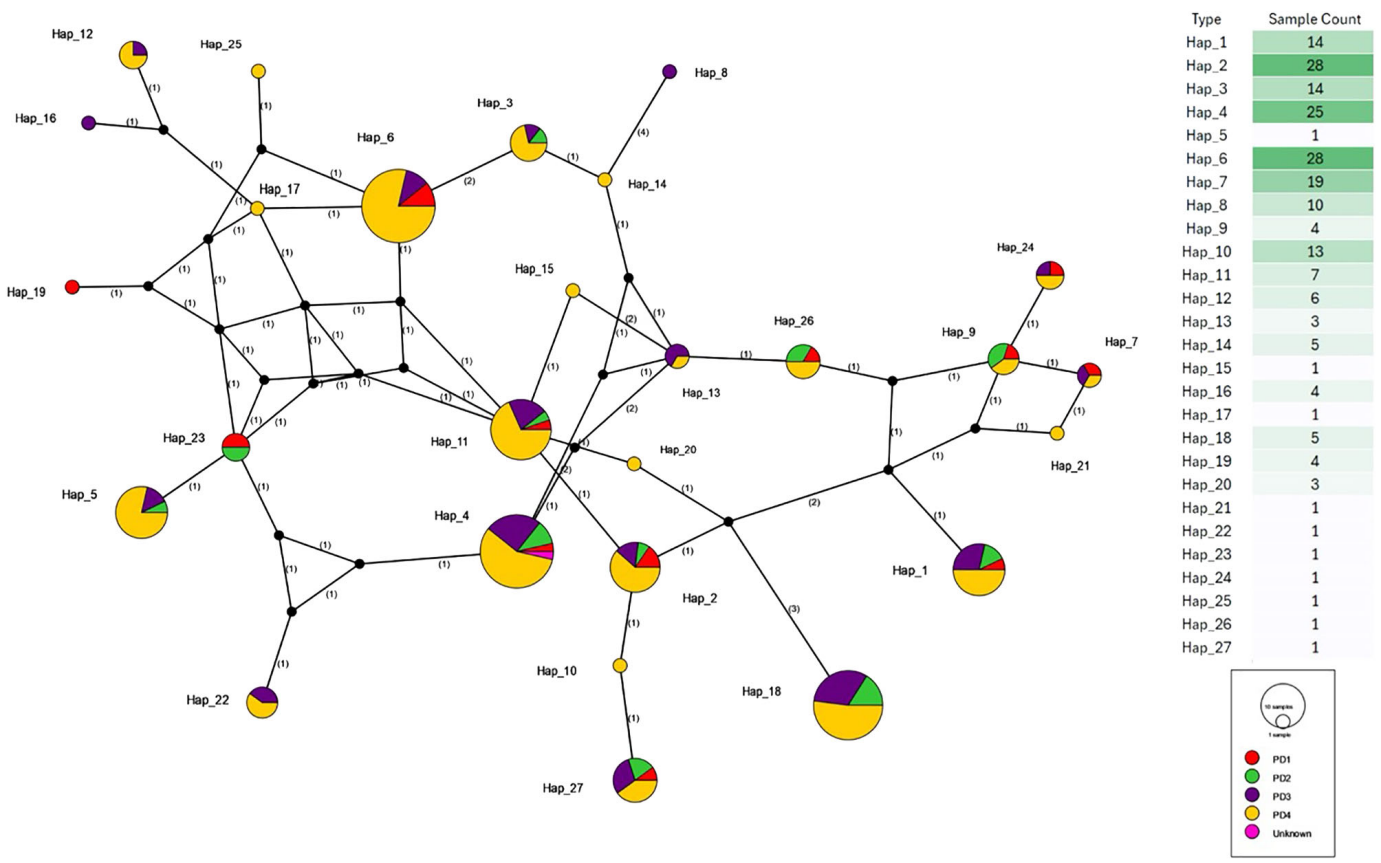
Haplotype network of *P. falciparum CelTOS* gene sequences categorised by parasite density. Nodes represent unique *CelTOS* haplotypes, scaled by frequency. Colours reflect parasite-density groups: red = PD1 (<1,000 parasites/μL), green = PD2 (1,000–9,999/μL), purple = PD3 (10,000–99,999/μL), yellow = PD4 (≥100,000/μL), and pink = unknown density. Central haplotypes (e.g., Hap_2, Hap_6, and Hap_10) include many high-density infections but also low- and moderate-density cases; consistent with the non-significant global χ² test (χ² = 61.7, df = 52, p = 0.167), these patterns do not support a strong, haplotype-specific association with elevated parasitaemia.

All 27 haplotypes were next examined according to parasitaemia category: PD1 (<1,000 parasites/µL), PD2 (1,000–9,999/µL), PD3 (10,000–99,999/µL), PD4 (≥100,000/µL), and Unknown. The density-stratified network (**Figure 8**) reveals that the most frequent haplotypes (Hap_2, Hap_4, Hap_6, Hap_7, Hap_1, Hap_3, and Hap_10) are also the most density-diverse, typically containing three or four parasitaemia classes. For example, Hap_6 (n = 28) is dominated by PD4 infections but includes smaller wedges of PD2 and PD3 (and occasionally PD1), while Hap_2 and Hap_4 show a similar pattern of PD4 predominance with contributions from lower-density categories. Hap_11 and Hap_23 also exhibit a broad mix of density classes despite more modest sample sizes.

At the opposite extreme, several rare peripheral haplotypes are density-narrow, being composed almost entirely of a single parasitaemia class. For instance, one small haplotype is exclusively PD1 (e.g., Hap_19), whereas others are almost entirely PD3 or PD4 (e.g., Hap_16, Hap_18). Because these haplotypes are represented by only one or a few isolates each, their apparent density specificity likely reflects stochastic sampling rather than strong biological specialisation.

Overall, the network shows broad overlap of density colours across the core of the graph. Combined with the non-significant chi-square test for independence between haplotype and parasitaemia category (**Figure 4**; χ² = 61.7, df = 52, p = 0.167), these patterns indicate that parasite density varies widely within individual haplotypes, and no single *CelTOS* lineage is uniquely associated with low or very high parasitaemia. Any association between *CelTOS* variation and peripheral density is therefore likely to be weak relative to host and environmental determinants.

### 3.12 Haplotype network analysis of *CelTOS* gene by geographic region

**Figure 9:**
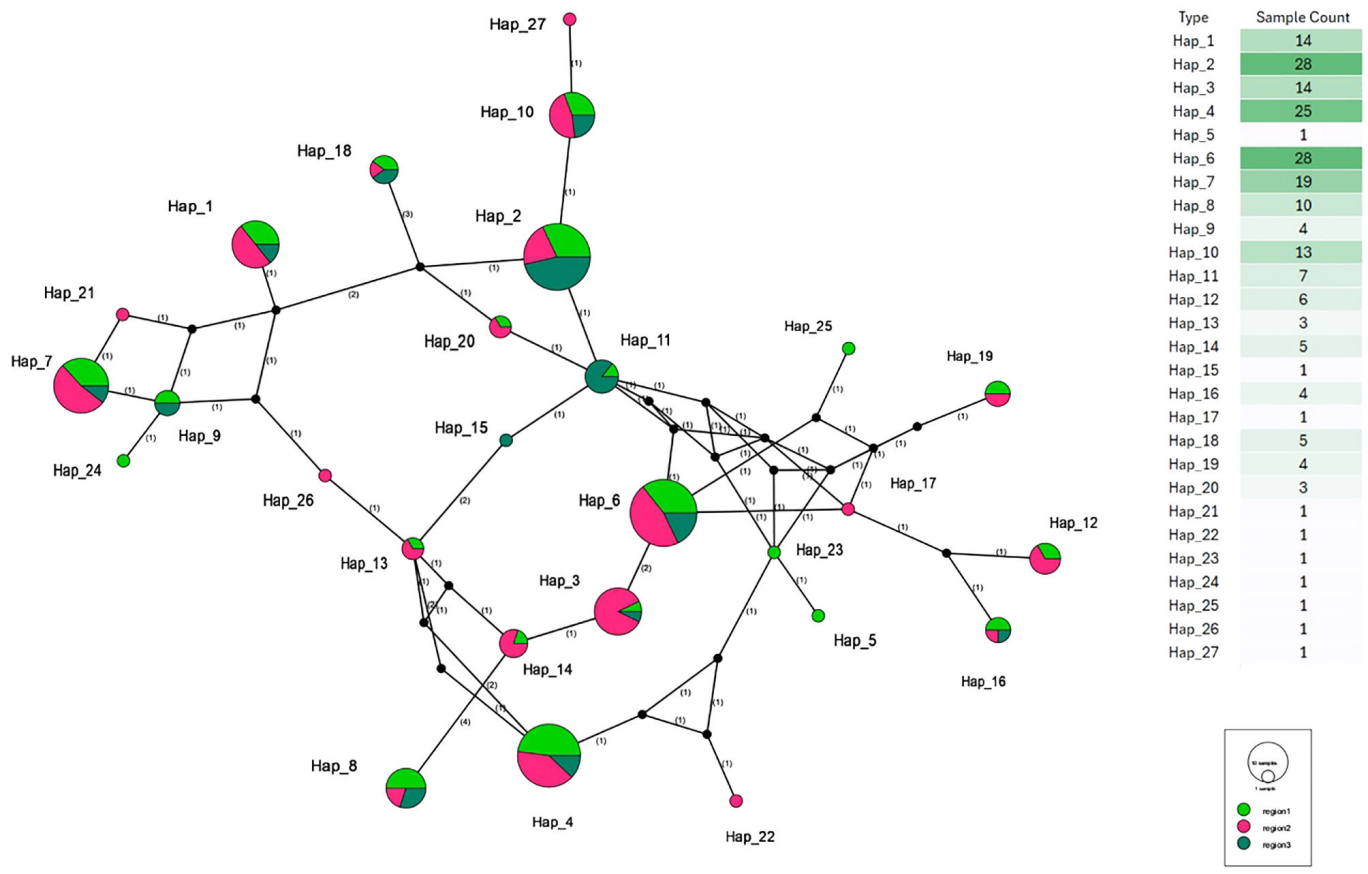
Haplotype network of *P. falciparum CelTOS* gene sequences categorised by geographic region within Jazan Province. Node colours represent sample origin: green = region 1, pink = region 2, dark green = region 3. Central haplotypes are composed of mixed-region isolates, suggesting strong interregional gene flow. Peripheral haplotypes show partial regional clustering, which may reflect localised transmission dynamics or recent introductions. Underlying region counts per haplotype are in **Supplementary File 6a** (Region).

We then examined the distribution of haplotypes across the three Jazan subregions (Region 1–3). The regional network (**Figure 9**) again shows a pronounced core–periphery structure. The most frequent haplotypes—Hap_2, Hap_4, Hap_6, Hap_7, Hap_1, Hap_3, Hap_10, and Hap_11—are also the most geographically diverse, typically containing isolates from two or all three regions. In particular, Hap_2, Hap_4, Hap_6, and Hap_11 all display substantial wedges from region 1, region 2, and region 3, indicating that these lineages are widely distributed across the province.

In contrast, several low-frequency haplotypes are region-restricted. Singletons such as Hap_5, Hap_15, Hap_17, Hap_21, Hap_24–27, and a few small clusters (e.g., Hap_8, Hap_16, and Hap_22) appear to derive from only one subregion, forming small mono-coloured nodes at the network periphery. These likely represent localised transmission chains or recent introductions that have not yet disseminated to other areas.

Despite the presence of these region-restricted satellites, the overall colour mixing across the network is high, with most mutational paths running through multiregional core haplotypes. This visual impression is consistent with the near-zero pairwise F_ST_ estimates and non-significant PERMANOVA results and supports a scenario of minimal geographic structuring of *CelTOS* diversity within Jazan, with most genetic variation occurring within rather than between regions.

### 3.13 Haplotype network analysis of the *CelTOS* gene by seasonal exploration

**Figure 10:**
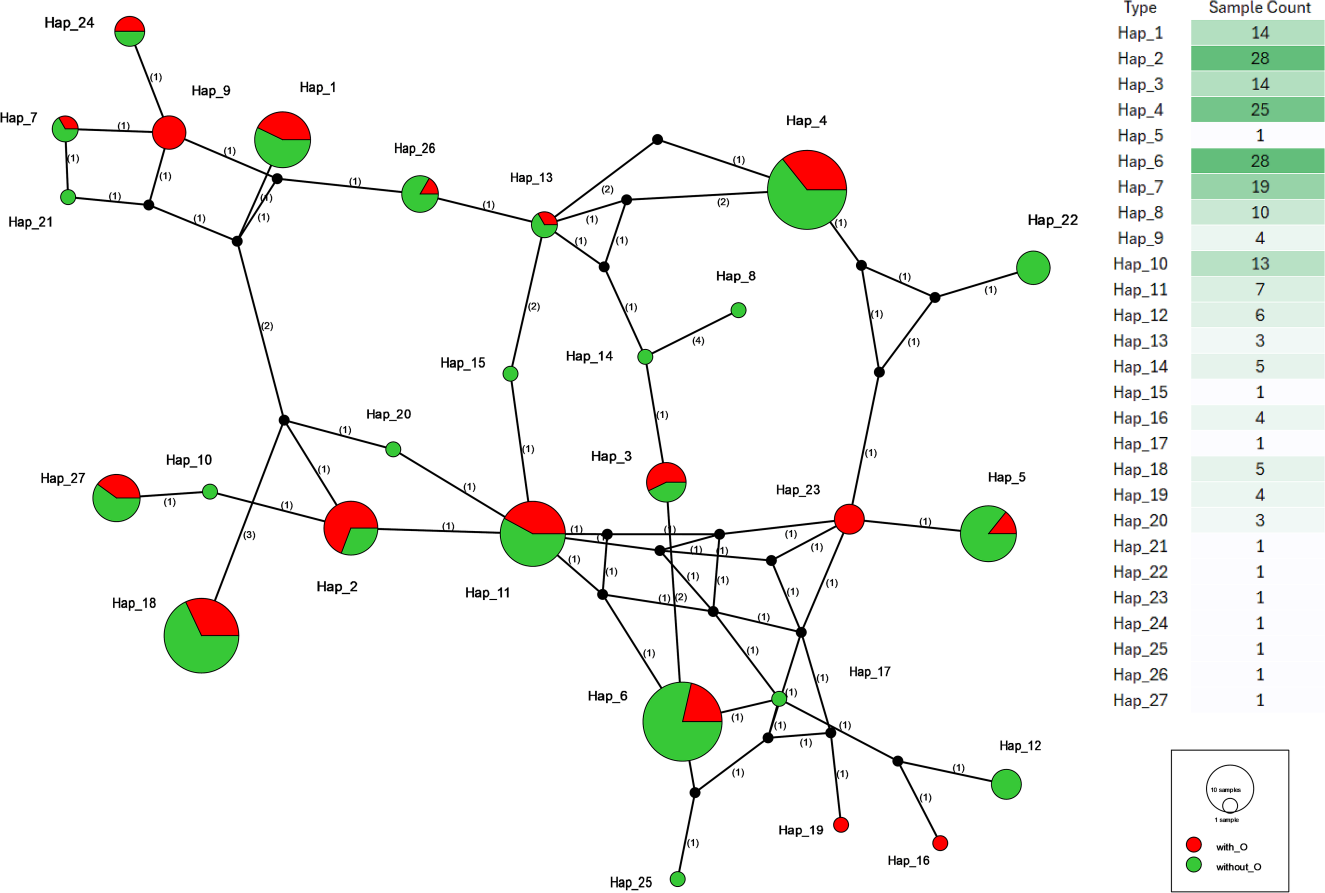
Haplotype network of *P. falciparum CelTOS* gene sequences categorised by transmission season. Colours indicate season of sample collection: red = winter (with_O), green = summer (without_O). Shared haplotypes (e.g., Hap_2 and Hap_6) appear in both seasons, suggesting year-round transmission. Season-specific haplotypes are concentrated in peripheral branches, pointing to possible temporal diversification or environmental selection across the transmission calendar.

Finally, we explored potential seasonal differences in *CelTOS* haplotype composition by classifying samples into winter (with_O) and summer (without_O) transmission periods. The season-stratified network (**Figure 10**) shows that most of the common core haplotypes—Hap_2, Hap_4, Hap_6, Hap_7, Hap_1, Hap_3, Hap_10, and Hap_11—contain isolates from both seasons, often in comparable proportions. For example, Hap_4 and Hap_6 each show large pies split between winter and summer wedges, indicating that these high-frequency lineages circulate throughout the year.

Some haplotypes, particularly among the rare peripheral types, are season-biased or season-specific. A few small nodes (e.g., Hap_21 and Hap_24) are composed entirely of winter isolates, whereas others (e.g., Hap_8, Hap_18, and Hap_20) are dominated by summer infections. However, these haplotypes each contribute only one or a handful of isolates, so their apparent seasonality probably reflects limited sampling rather than strict seasonal restriction.

Overall, the backbone of the network is formed by season-mixed core haplotypes, with season-skewed nodes confined to the periphery. This pattern suggests that while individual *CelTOS* haplotypes may fluctuate in frequency between winter and summer, the underlying haplotype pool is largely shared across seasons, consistent with ongoing, year-round transmission of a common set of lineages rather than complete seasonal turnover.

## 4 Discussion

This study provides a comprehensive assessment of *P. falciparum CelTOS* gene diversity and population structure in the malaria-endemic Jazan Province of Saudi Arabia. Through an integrated approach combining haplotype networks, population-genetic analyses, and regression modelling, we demonstrate that parasite populations in this region are genetically diverse, highly interconnected, and influenced by demographic, environmental, and cross-border factors. The findings highlight the evolutionary and epidemiological mechanisms shaping parasite persistence in this critical border region between Saudi Arabia and Yemen.

Building on these network-level insights, we incorporated formal population-genetic tests on the final molecular dataset (n = 202 sequences; 726 ungapped coding sites). Neutrality and demographic-fit summaries (DnaSP), mismatch distributions (SSD and raggedness), recombination testing (PHI test, p = 1.438 × 10^−5^), recombination-aware codon-level selection scans (SLAC/FEL/MEME/FUBAR via Datamonkey), and population-structure analyses (pairwise F_ST_, PCoA/PERMANOVA, and hierarchical AMOVA) collectively support a picture of limited differentiation across region and nationality, high haplotype but low nucleotide diversity, and no strong evidence for pervasive positive selection after explicitly accounting for recombination. The unimodal mismatch distribution with low SSD (0.029) and raggedness (0.0487) is compatible with a past demographic expansion, whereas mostly non-significant neutrality indices and near-zero F_ST_ values indicate that any current departures from neutrality or geographic structure are modest. Full mismatch, neutrality, selection, and structure outputs (including DnaSP mismatch/neutrality exports, the global diversity summary, Datamonkey selection reports, and AMOVA result files) are provided and explained in **Supplementary Files 3a, b, 4a, 5a, b, 7**, **4a, 5a, b, 7**.

### 4.1 Shared haplotypes and population connectivity

Dominant *CelTOS* haplotypes (e.g., Hap_2, Hap_6, and Hap_7) were shared across nationality groups, parasite-density categories, geographic regions, and seasons, suggesting a core set of lineages that sustain year-round transmission. These central haplotypes appear to act as genetic hubs linking multiple peripheral variants, indicating frequent gene flow among subpopulations and the absence of deep population stratification.

This high degree of haplotype sharing is consistent with a panmictic population structure facilitated by human movement and overlapping vector ranges (Conway et al., 1999; Mobegi et al., 2012). Similar interconnectivity has been observed in *P. falciparum* populations from other border and migration-prone regions such as Papua New Guinea and Senegal, where gene flow persists despite physical or ecological barriers (Barry et al., 2015; Wotodjo et al., 2023). Within the Arabian Peninsula, previous reports describe extensive diversity and genetic connectivity in Jazan, likely driven by continuous importation of malaria from neighbouring Yemen through labour migration and population mobility (Al-Hamidhi et al., 2014; Bamaga et al., 2014; El Hassan et al., 2015; Al-Mekhlafi et al., 2021; Elagali et al., 2024). These findings reinforce the need for cross-border coordination in malaria elimination efforts, emphasising that localised control alone will not be sufficient in such interconnected settings.

The intermixing observed here underscores the necessity of designing cross-cutting control strategies that address all population groups and districts simultaneously, particularly given the documented cross-border malaria transmission between Saudi Arabia and Yemen (Bamaga et al., 2014; Elagali et al., 2024). Previous studies have highlighted that multiple genetic population studies in the Arabian Peninsula reported large extent of diversity and parasite genetic structure in the Jazan region maybe due to a constant influx of imported malaria infections into the region from surrounding areas in Yemen as infected skilled workers, which supports our findings of high genetic connectivity across demographic groups (Al-Hamidhi et al., 2014; El Hassan et al., 2015; Al-Mekhlafi et al., 2021).

Formal structure tests were concordant with these network patterns: pairwise F_ST_ values between nationalities and between regions were generally low, and AMOVA attributed only a small fraction of total variance to among-population components. In parallel, the overall mismatch distribution was unimodal and fit a sudden-expansion model, further supporting recent expansion with extensive gene flow. Together, these signals are compatible with a largely panmictic parasite population at the *CelTOS* locus in Jazan.

### 4.2 *CelTOS* gene diversity in regional context

Our identification of 27 *CelTOS* haplotypes with substantial diversity is consistent with global patterns reported for this antigen. Previous studies have shown that *CelTOS* displayed a moderate frequency of unique haplotypes at 33.9% across diverse populations, suggesting that this antigen maintains considerable polymorphism despite its functional importance in sporozoite invasion. The moderate diversity we observed aligns with findings from transmission-blocking vaccine candidate assessments, where *CelTOS* showed intermediate levels of variation compared with other antigens (Ciubotariu et al., 2024).

The star-like network topology with central hub haplotypes connected to peripheral variants by single mutational steps is characteristic of populations that have undergone demographic expansion and frequent gene flow, with only localised selection acting on a subset of sites. This pattern has been observed in other *P. falciparum* antigens and suggests that *CelTOS* may be under immune pressure while maintaining functional constraints that limit extreme diversification (Neafsey et al., 2008; Tandoh et al., 2021).

Diversity indices (π, Hd, θW) were consistently low-to-moderate across region and nationality strata and tightly clustered, indicating broadly similar standing variation across groups. After accounting for significant recombination (PHI p = 1.438×10^−5^), codon-level scans did not identify robust, concordant evidence for pervasive diversifying selection at *CelTOS*. These findings suggest that, whereas *CelTOS* exhibits notable haplotypic richness, its protein-coding variation may be shaped by a balance of functional constraint, episodic or weak selection, and frequent gene flow. For vaccine design, these results argue for formulations that cover the dominant haplotypes but do not currently indicate strong, localised immune escape at this locus.

### 4.3 Selection analyses on *CelTOS*

Selective-pressure testing revealed a gene-wide dN/dS ratio (ω) of approximately 0.36, indicating that *CelTOS* evolves under predominant purifying selection, consistent with its essential role in parasite cell traversal. However, codon-based analyses (MEME, FEL, and SLAC) identified a limited set of sites under episodic diversifying selection, mainly within solvent-exposed or predicted immunogenic regions of the protein. This pattern suggests that localised immune-mediated diversification occurs on a largely conserved structural backbone. The coexistence of strong functional constraint with focal diversifying selection is typical of surface-exposed *P. falciparum* antigens such as CSP and AMA1, which balance immune escape with the need to maintain core function. Detailed codon-level selection estimates for all models are provided in **Supplementary File 7**.

### 4.4 Subregional uniformity and subtle geographic structure

Despite local environmental variation, haplotype diversity remained high across all three Jazan subregions (Hd 0.89–0.92) whereas nucleotide diversity was low (π ≈ 0.005–0.006; **Figure 3**). Neutrality indices (Tajima’s D, Fu & Li’s D*, and Fu & Li’s F*) were positive in every subregion (**Figure 3**), but none exceeded conventional significance thresholds, indicating no strong departure from neutrality at *CelTOS* in any part of the province. The combination of high haplotype but low nucleotide diversity is compatible with a history of population expansion followed by relatively stable transmission.

Principal coordinate analysis (PCoA) based on pairwise genetic distances showed extensive overlap among subregions (**Figure 5**). The first two axes captured 70.2% of the total variance (PC1: 37.7%; PC2: 32.5%), and PERMANOVA indicated only weak, non-significant differentiation by region (pseudo-F = 1.75, R² = 0.017, p = 0.078). Pairwise F_ST_ estimates among subregions were essentially zero and slightly negative (approximately −0.005 to 0.000; **Figure 6**), and AMOVA attributed nearly all genetic variance to within-population components. Collectively, these results support a largely panmictic parasite population at the *CelTOS* locus, with at most very subtle geographic heterogeneity that is not statistically robust.

Any residual structure is therefore more likely to reflect shortterm micro-ecological differences and local transmission intensity than long-term isolation of distinct parasite subpopulations. Similar microstructural patterns have been reported in malaria-endemic regions approaching elimination, where residual transmission becomes increasingly focal (Anstey et al., 2021).

### 4.5 Demographic versus geographic contributions

In keeping with the largely panmictic population structure, the final regression analyses indicated that demographic factors exerted at most modest effects on very high parasitaemia. In the multivariable logistic regression model contrasting PD4 with PD1–PD3 (Section 4.3; **Table 2**), older age and non-Saudi nationality were associated with slightly higher odds of very high parasitaemia (age: OR = 1.03; 95% CI 1.00–1.06; nationality: OR = 2.21; 95% CI 1.02–4.81), whereas sex and region were not significant predictors. Exploratory models using alternative parasitaemia cut-points and ordinal logistic regression (**Supplementary File 4b**) yielded qualitatively similar results, reinforcing that demographic influences on parasite density are real but small and imprecisely estimated in this dataset. Overall, these findings suggest that *CelTOS* diversity and haplotype structure cut across simple demographic strata and that host-related contributions to parasite genotypes or parasitaemia are likely to be modest relative to ecological and health-system determinants, particularly given the available sample size.

### 4.6 Border dynamics and cross-border transmission

The genetic connectivity across nationality groups reflects the cross-border nature of malaria transmission in Jazan, sustained by human migration and ecological continuity with Yemen. Shared haplotypes between Saudi and non-Saudi patients confirm that *P. falciparum* populations are not geographically isolated but form a continuous genetic network spanning the border.

National surveillance reports support this conclusion, showing that most malaria cases in Jazan involve Yemeni nationals, followed by Saudis. Similar cross-border gene flow and persistence of residual transmission have been documented in other elimination settings, where human mobility can undermine local control efforts (Tatem et al., 2017; Lynch et al., 2008). For Jazan, effective elimination will require joint Saudi–Yemeni control initiatives, incorporating synchronised vector control and genomic surveillance to trace parasite flow and evaluate intervention outcomes.

### 4.7 Recombination screening

Network reticulation and formal recombination testing indicate that the *CelTOS* locus in this dataset does not evolve under a strictly tree-like process. The PHI test provided strong evidence for recombination in the alignment (p = 1.438 × 10^−5^), consistent with the presence of multiple median vectors and alternative connections in the haplotype network. Because unmodelled recombination can inflate false-positive signals of selection, we relied on Datamonkey’s recombination-aware implementations of SLAC, FEL, MEME, and FUBAR when interpreting site-specific selection signals. Our demographic and selection inferences are therefore based on analytical frameworks that explicitly accommodate recombination.

### 4.8 Implications for control and surveillance

Taken together, these findings indicate that *P. falciparum* in Jazan functions as a single, interconnected metapopulation, with limited spatial structure but marked demographic and seasonal influences. Control programs must therefore adopt an integrated regional approach, incorporating cross-border coordination, molecular surveillance, and year-round interventions to prevent reintroduction.

Haplotype-based analyses provide a valuable molecular complement to epidemiological monitoring, enabling the detection of introduction events, emergence of new variants, or potential vaccine escape haplotypes. Given its moderate diversity and partial conservation, *CelTOS* is both a promising vaccine target and a molecular marker for transmission monitoring. Integrating these genetic tools into Saudi Arabia’s national elimination strategy will enhance early detection and allow precision targeting of residual foci.

### 4.9 Parasitaemia and *CelTOS* haplotype associations

Across *CelTOS* haplotypes, mean parasitaemia levels spanned the full ordinal range (1–4), with some common haplotypes clustering toward higher-density infections and others showing more moderate or low values (**Figure 4**). However, the global association between categorical parasitaemia and haplotype composition was not statistically significant (χ² = 61.7, df = 52, p = 0.167). Thus, while certain haplotypes appear descriptively enriched among high-parasitaemia infections, these differences cannot be interpreted as robust, haplotype-specific effects with the current sample size.

To formally quantify these relationships, we fitted a multivariable logistic regression model contrasting very high parasitaemia (PD4) with lower categories (PD1–PD3) (Methods; **Table 2**). In this model, older age and non-Saudi nationality were associated with modestly higher odds of very high parasitaemia (age: OR = 1.03; 95% CI 1.00–1.06; nationality: OR = 2.21; 95% CI 1.02–4.81), whereas region of residence and sex were not significant predictors. Exploratory models using alternative parasitaemia cut-points and ordinal logistic regression gave qualitatively similar conclusions and are summarised in **Supplementary File 4b**. These findings, together with the non-significant haplotype-level χ² test, suggest that host and contextual factors such as age and nationality exert a stronger influence on peripheral parasite density than *CelTOS* polymorphism alone, although larger, multilocus datasets will be needed to confirm these patterns.

Biologically, it remains plausible that *CelTOS* variants could influence parasite fitness indirectly, for example by affecting sporozoite traversal and early liver-stage success, which could in turn modulate downstream blood-stage parasite burdens (Jimah et al., 2016). Our data, however, do not provide definitive evidence that particular *CelTOS* haplotypes are intrinsically “high-parasitemia” or “lowparasitemia” variants. Host immunity, prior exposure, co-infections, and other parasite loci are likely to exert stronger influence on clinical parasite density than *CelTOS* polymorphism alone (Mackinnon et al., 2000), and larger, multi-locus studies will be required to disentangle these effects.

### 4.10 Seasonality and temporal evolution

The season-stratified haplotype network provides a first glimpse of temporal dynamics at the *CelTOS* locus. Most common core haplotypes (e.g., Hap_2, Hap_6, and Hap_7) contain isolates from both winter and summer, indicating a largely shared backbone of lineages that circulate throughout the year (**Figure 10**). At the periphery of the network, a small number of low-frequency haplotypes appear season-skewed or season-specific, often linked to the cores by single mutational steps. These peripheral patterns are compatible with short-term diversification, seasonal fluctuations in transmission intensity, or stochastic sampling, but they are based on few observations and were not formally tested with time-series models. Broadly similar seasonal fluctuations in parasite genetic composition have been reported in other malaria-endemic settings (Konaté et al., 1999; Tessema et al., 2019).

The persistence of major haplotypes across seasons supports the presence of perennial parasite reservoirs sustaining transmission throughout the year, which is critical for understanding elimination challenges in the region. Even if some minor haplotypes fluctuate in frequency between winter and summer, the overall picture is one of a shared lineage pool modulated by seasonal ecology rather than complete turnover of parasite genotypes. This temporal heterogeneity underscores the importance of sustained year-round intervention efforts and reinforces the utility of molecular markers for evaluating seasonal transmission patterns, particularly in regions like Jazan where targeted Southern-region control and enhanced surveillance are central to pre-elimination strategies.

### 4.11 Study limitations

Although this study provides detailed insights into *CelTOS* diversity, it is constrained by several limitations.

Single-locus analysis: Whole-genome or multilocus approaches could uncover additional structure and selection not captured here.

Temporal coverage: Sampling represents limited seasons; extended longitudinal sampling would clarify whether seasonal haplotype shifts persist over time.

Functional implications: The biological impact of *CelTOS* polymorphisms remains unknown; functional assays and immuno-epidemiological analyses are needed to determine their influence on invasion, immunity, and vaccine efficacy.

## 5 Conclusion

This work reveals a complex but coherent portrait of *P. falciparum* evolution in Jazan Province. The dominance of shared *CelTOS* haplotypes across demographic and ecological gradients underscores extensive gene flow and sustained transmission, whereas localised and seasonal variants hint at adaptive microevolution operating at finer spatial and temporal scales. Formal population-genetic analyses point to high haplotype but low nucleotide diversity, near-panmictic structure at the subregional level and predominantly purifying selection acting on *CelTOS*, with only limited evidence for episodic diversification at a few putative immune-exposed sites. These findings have direct implications for malaria elimination and vaccine design, emphasising the need for coordinated cross-border interventions, continuous genomic surveillance and broadly protective vaccine formulations targeting conserved *P. falciparum* antigens such as *CelTOS*, while also monitoring for the emergence of new variants in this epidemiologically complex border region.

## Data Availability

The CelTOS nucleotide sequences generated in this study are available in GenBank under accession numbers PX802477–PX802678. A direct NCBI record link is: https://www.ncbi.nlm.nih.gov/nuccore/PX802477 (readers can replace PX802477 in the URL with any accession in the range up to PX802678 to view the corresponding sequence record). The CelTOS codon alignment (SF2a), linked de-identified clinical and demographic traits (SF2b), downstream analysis outputs (SF3a–SF7), and all custom Python scripts used for analyses and figure generation are available as Supplementary Material, which is hosted with the published article and deposited to Figshare with assigned DOIs.
